# (2,2′-Bipyridyl-κ^2^ *N*,*N*′)tetra­kis(4-methyl­benzoato-κ*O*)manganese(II)

**DOI:** 10.1107/S160053680800994X

**Published:** 2008-04-16

**Authors:** Wen-Dong Song, Hao Wang, De-Yun Ma

**Affiliations:** a College of Science, Guang Dong Ocean University, Zhan Jiang 524088, People’s Republic of China; b College of Chemistry, South China University of Technology, Guangzhou 510640, People’s Republic of China

## Abstract

In the title mononuclear complex, [Mn(C_8_H_7_O_2_)_4_(C_10_H_8_N_2_)], the Mn^II^ atom lies on a twofold rotation axis and has a distorted octa­hedral coordination geometry defined by four O atoms from four 4-methyl­benzoate ligands and two N atoms from one 2,2′-bipyridyl ligand. The crystal structure is stabilized by inter­molecular hydrogen bonds and π–π stacking inter­actions [the centroid–centroid distance between the parallel pyridyl ring of a 2,2′-bipyridyl and benzene ring of a 4-methylbenzoic group of a neighboring complex is 3.839 (2) Å].

## Related literature

For related literature, see: Song *et al.* (2007 ▶).
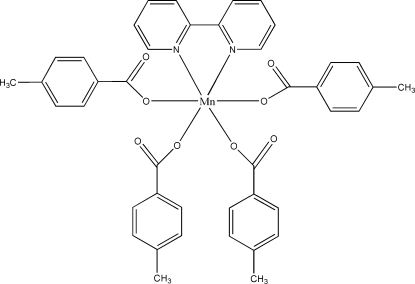


## Experimental

### 

#### Crystal data

[Mn(C_8_H_7_O_2_)_4_(C_10_H_8_N_2_)]
*M* *_r_* = 753.68
Orthorhombic, 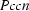

*a* = 13.6159 (2) Å
*b* = 14.2585 (2) Å
*c* = 19.5732 (3) Å
*V* = 3799.99 (10) Å^3^
*Z* = 4
Mo *K*α radiation
μ = 0.40 mm^−1^
*T* = 273 (2) K
0.20 × 0.16 × 0.11 mm

#### Data collection

Bruker APEXII area-detector diffractometer
Absorption correction: multi-scan (*SADABS*; Sheldrick, 1996 ▶) *T*_min_ = 0.93, *T*_max_ = 0.96
30915 measured reflections
4378 independent reflections
2297 reflections with *I* > 2σ(*I*)
*R*_int_ = 0.070

#### Refinement

*R*[*F* ^2^ > 2σ(*F* ^2^)] = 0.047
*wR*(*F* ^2^) = 0.131
*S* = 1.01
4378 reflections
243 parameters
H-atom parameters constrained
Δρ_max_ = 0.22 e Å^−3^
Δρ_min_ = −0.24 e Å^−3^

### 

Data collection: *APEX2* (Bruker, 2004 ▶); cell refinement: *SAINT* (Bruker, 2004 ▶); data reduction: *SAINT*; program(s) used to solve structure: *SHELXS97* (Sheldrick, 2008 ▶); program(s) used to refine structure: *SHELXL97* (Sheldrick, 2008 ▶); molecular graphics: *XP* in *SHELXTL* (Sheldrick, 2008 ▶); software used to prepare material for publication: *SHELXTL*.

## Supplementary Material

Crystal structure: contains datablocks I, global. DOI: 10.1107/S160053680800994X/bg2175sup1.cif

Structure factors: contains datablocks I. DOI: 10.1107/S160053680800994X/bg2175Isup2.hkl

Additional supplementary materials:  crystallographic information; 3D view; checkCIF report

## Figures and Tables

**Table 1:** Hydrogen-bond geometry (Å, °)

*D*—H⋯*A*	*D*—H	H⋯*A*	*D*⋯*A*	*D*—H⋯*A*
O4—H4*A*⋯O2^i^	0.82	1.68	2.477 (2)	164

Symmetry code: (i) 
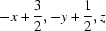
.

**Fig. 1.**
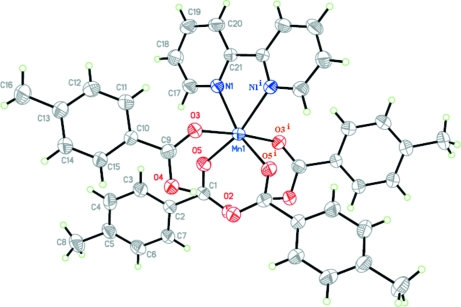
The structure of (I), showing the atomic numbering scheme. Non-H atoms are shown as 30% probability displacement ellipsoids.

## supplementary crystallographic information

### Comment

In the structural investigation of 4-methylbenzate complexes, it has been found that the 4-methylbenzoic acid functions as a multidentate ligand [Song *et al.* (2007)], with versatile binding and coordination modes. In this paper, we report the crystal structure of the title compound, (I), a new Mn complex obtained by the reaction of 4-methylbenzoic acid, 2,2'-bipyridyl and manganese chloride in alkaline aqueous solution.

As illustrated in Figure 1, the Mn^II^ atom lies on a centre of symmetry, and is surrounded by distorted octahedral environment defined by four carboxyl O atoms from four monodentate 4-methylbenzate ligands and two N atoms from one 2,2'-bipyridyl ligand. The packing is governed by a O—H···O hydrogen bond (Table 1) and a weak π-π stacking interaction between the parallel pyridyl ring of a 2,2'-bipyridyl and a phenyl ring of 4-methylbenzoic group of neighboring complexes (1.5-x, 0.5-y, z), with a centroid to centroid distance of 3.839 (2) Å.

### Experimental

A mixture of manganese chloride(1 mmol), 4-methylbenzoic acid (1 mmol), 2,2'-bipyridyl(1 mmol), NaOH (1.5 mmol) and H_2_O (12 ml) was placed in a 23 ml Teflon reactor, which was heated to 433 K for three days and then cooled to room temperature at a rate of 10 K h^-1^. The crystals obtained were washed with water and dryed in air.

### Refinement

H atoms were placed at calculated positions and were treated as riding on the parent C atoms with C—H = 0.93 - 0.97 Å, N—H = 0.86 Å, and with U_iso_(H) = 1.2 U_eq_(C, N).

### Crystal data

**Table:**

[Mn(C_8_H_7_O_2_)_4_(C_10_H_8_N_2_)]	*F*_000_ = 1572
*M**_r_* = 753.68	*D*_x_ = 1.317 Mg m^−^^3^
Orthorhombic, *P**c**c**n*	Mo *K*α radiation λ = 0.71073 Å
Hall symbol: -P 2ab 2ac	Cell parameters from 3500 reflections
*a* = 13.6159 (2) Å	θ = 1.4–28.0º
*b* = 14.2585 (2) Å	µ = 0.40 mm^−^^1^
*c* = 19.5732 (3) Å	*T* = 273 (2) K
*V* = 3799.99 (10) Å^3^	Block, colourless
*Z* = 4	0.20 × 0.16 × 0.11 mm

### Data collection

**Table:**

Bruker APEXII area-detector diffractometer	4378 independent reflections
Radiation source: fine-focus sealed tube	2297 reflections with *I* > 2σ(*I*)
Monochromator: graphite	*R*_int_ = 0.071
*T* = 273(2) K	θ_max_ = 27.5º
φ and ω scans	θ_min_ = 2.1º
Absorption correction: multi-scan(SADABS; Sheldrick, 1996)	*h* = −17→17
*T*_min_ = 0.93, *T*_max_ = 0.96	*k* = −18→18
30915 measured reflections	*l* = −25→25

### Refinement

**Table:**

Refinement on *F*^2^	Secondary atom site location: difference Fourier map
Least-squares matrix: full	Hydrogen site location: inferred from neighbouring sites
*R*[*F*^2^ > 2σ(*F*^2^)] = 0.047	H-atom parameters constrained
*wR*(*F*^2^) = 0.131	*w* = 1/[σ^2^(*F*_o_^2^) + (0.0559*P*)^2^ + 0.3551*P*] where *P* = (*F*_o_^2^ + 2*F*_c_^2^)/3
*S* = 1.01	(Δ/σ)_max_ = 0.002
4378 reflections	Δρ_max_ = 0.22 e Å^−^^3^
243 parameters	Δρ_min_ = −0.24 e Å^−^^3^
Primary atom site location: structure-invariant direct methods	Extinction correction: none

### Special details

**Table:**

Geometry. All esds (except the esd in the dihedral angle between two l.s. planes) are estimated using the full covariance matrix. The cell esds are taken into account individually in the estimation of esds in distances, angles and torsion angles; correlations between esds in cell parameters are only used when they are defined by crystal symmetry. An approximate (isotropic) treatment of cell esds is used for estimating esds involving l.s. planes.
Refinement. Refinement of F^2^ against ALL reflections. The weighted R-factor wR and goodness of fit S are based on F^2^, conventional R-factors R are based on F, with F set to zero for negative F^2^. The threshold expression of F^2^ > 2sigma(F^2^) is used only for calculating R-factors(gt) etc. and is not relevant to the choice of reflections for refinement. R-factors based on F^2^ are statistically about twice as large as those based on F, and R- factors based on ALL data will be even larger.

### Fractional atomic coordinates and isotropic or equivalent isotropic displacement parameters (Å^2^)

**Table:**

	*x*	*y*	*z*	*U*_iso_*/*U*_eq_	
C1	0.8584 (2)	0.14977 (18)	0.04194 (12)	0.0562 (7)	
C2	0.95641 (18)	0.13966 (17)	0.00816 (12)	0.0540 (6)	
C3	1.0397 (2)	0.17677 (19)	0.03729 (13)	0.0653 (7)	
H3	1.0347	0.2093	0.0783	0.078*	
C4	1.1305 (2)	0.1667 (2)	0.00678 (15)	0.0748 (8)	
H4	1.1858	0.1922	0.0276	0.090*	
C5	1.1402 (2)	0.1191 (2)	−0.05468 (15)	0.0687 (8)	
C6	1.0571 (2)	0.0818 (2)	−0.08347 (14)	0.0721 (8)	
H6	1.0621	0.0485	−0.1242	0.087*	
C7	0.9662 (2)	0.09274 (19)	−0.05331 (13)	0.0662 (7)	
H7	0.9108	0.0682	−0.0746	0.079*	
C8	1.2399 (2)	0.1071 (3)	−0.08752 (16)	0.0941 (11)	
H8A	1.2416	0.0489	−0.1122	0.141*	
H8B	1.2896	0.1066	−0.0528	0.141*	
H8C	1.2517	0.1581	−0.1185	0.141*	
C9	0.9071 (2)	0.39242 (17)	0.11458 (13)	0.0563 (7)	
C10	1.01048 (19)	0.40889 (18)	0.13322 (12)	0.0532 (6)	
C11	1.0399 (2)	0.3921 (2)	0.19970 (13)	0.0752 (8)	
H11	0.9946	0.3709	0.2317	0.090*	
C12	1.1359 (2)	0.4068 (3)	0.21850 (15)	0.0940 (11)	
H12	1.1546	0.3943	0.2633	0.113*	
C13	1.2057 (2)	0.4397 (3)	0.17272 (15)	0.0834 (9)	
C14	1.1752 (2)	0.4553 (2)	0.10613 (14)	0.0757 (8)	
H14	1.2203	0.4767	0.0740	0.091*	
C15	1.07983 (19)	0.4399 (2)	0.08677 (13)	0.0645 (7)	
H15	1.0614	0.4504	0.0417	0.077*	
C16	1.3096 (2)	0.4584 (4)	0.19472 (18)	0.1358 (17)	
H16A	1.3107	0.4735	0.2425	0.204*	
H16B	1.3356	0.5101	0.1690	0.204*	
H16C	1.3489	0.4036	0.1867	0.204*	
C17	0.9252 (2)	0.1655 (2)	0.25483 (13)	0.0669 (8)	
H17	0.9504	0.1479	0.2126	0.080*	
C18	0.9816 (2)	0.1503 (2)	0.31183 (14)	0.0731 (8)	
H18	1.0436	0.1235	0.3081	0.088*	
C19	0.9451 (2)	0.1753 (2)	0.37414 (14)	0.0765 (9)	
H19	0.9820	0.1665	0.4136	0.092*	
C20	0.8529 (2)	0.2136 (2)	0.37739 (13)	0.0683 (8)	
H20	0.8262	0.2297	0.4195	0.082*	
C21	0.79986 (17)	0.22837 (15)	0.31865 (11)	0.0482 (6)	
Mn1	0.7500	0.2500	0.16384 (2)	0.05147 (19)	
N1	0.83575 (14)	0.20438 (14)	0.25699 (9)	0.0528 (5)	
O2	0.78417 (13)	0.12153 (14)	0.00966 (9)	0.0733 (6)	
O3	0.84470 (13)	0.37396 (13)	0.15885 (8)	0.0639 (5)	
O4	0.88686 (13)	0.39944 (15)	0.04991 (9)	0.0709 (5)	
H4A	0.8276	0.3932	0.0442	0.106*	
O5	0.85649 (12)	0.18654 (14)	0.10029 (8)	0.0678 (5)	

### Atomic displacement parameters (Å^2^)

**Table:**

	*U*^11^	*U*^22^	*U*^33^	*U*^12^	*U*^13^	*U*^23^
C1	0.0513 (17)	0.0700 (18)	0.0472 (14)	−0.0002 (14)	0.0052 (13)	0.0032 (12)
C2	0.0490 (16)	0.0645 (16)	0.0486 (13)	−0.0017 (13)	0.0049 (13)	−0.0004 (12)
C3	0.0604 (19)	0.0755 (19)	0.0600 (16)	−0.0043 (15)	0.0059 (14)	−0.0106 (13)
C4	0.0501 (18)	0.096 (2)	0.078 (2)	−0.0066 (16)	0.0084 (15)	−0.0094 (17)
C5	0.0552 (19)	0.085 (2)	0.0658 (17)	0.0056 (16)	0.0149 (15)	0.0032 (15)
C6	0.070 (2)	0.087 (2)	0.0592 (16)	0.0016 (17)	0.0170 (16)	−0.0097 (14)
C7	0.0585 (19)	0.085 (2)	0.0555 (15)	−0.0070 (15)	0.0102 (14)	−0.0086 (14)
C8	0.064 (2)	0.125 (3)	0.093 (2)	0.0086 (19)	0.0257 (18)	0.0028 (19)
C9	0.0590 (18)	0.0595 (16)	0.0503 (15)	−0.0025 (14)	−0.0041 (14)	0.0003 (12)
C10	0.0432 (16)	0.0671 (17)	0.0493 (14)	−0.0028 (13)	−0.0035 (12)	0.0007 (12)
C11	0.061 (2)	0.109 (2)	0.0553 (17)	−0.0080 (17)	−0.0025 (15)	0.0113 (15)
C12	0.069 (2)	0.158 (3)	0.0550 (17)	−0.010 (2)	−0.0133 (17)	0.0166 (19)
C13	0.0523 (19)	0.131 (3)	0.0671 (19)	−0.0014 (19)	−0.0081 (16)	0.0071 (17)
C14	0.0509 (18)	0.115 (2)	0.0611 (17)	−0.0078 (17)	0.0002 (15)	0.0089 (15)
C15	0.0491 (17)	0.089 (2)	0.0557 (15)	−0.0053 (15)	−0.0029 (14)	0.0112 (13)
C16	0.053 (2)	0.262 (5)	0.093 (3)	−0.020 (3)	−0.016 (2)	0.023 (3)
C17	0.0578 (18)	0.087 (2)	0.0556 (16)	0.0139 (16)	0.0053 (14)	−0.0018 (13)
C18	0.0575 (18)	0.093 (2)	0.0689 (18)	0.0218 (16)	−0.0041 (16)	0.0050 (15)
C19	0.066 (2)	0.106 (2)	0.0577 (17)	0.0165 (18)	−0.0163 (15)	0.0040 (15)
C20	0.068 (2)	0.091 (2)	0.0464 (15)	0.0106 (17)	−0.0047 (14)	−0.0017 (13)
C21	0.0472 (15)	0.0535 (15)	0.0438 (12)	−0.0031 (11)	−0.0006 (11)	−0.0001 (10)
Mn1	0.0442 (3)	0.0715 (4)	0.0387 (3)	−0.0011 (3)	0.000	0.000
N1	0.0456 (13)	0.0664 (13)	0.0463 (11)	0.0021 (11)	0.0025 (10)	0.0006 (9)
O2	0.0498 (12)	0.1187 (16)	0.0513 (10)	−0.0112 (11)	0.0035 (9)	−0.0116 (10)
O3	0.0559 (12)	0.0845 (13)	0.0513 (10)	−0.0149 (10)	0.0057 (9)	−0.0031 (9)
O4	0.0500 (12)	0.1085 (15)	0.0542 (11)	−0.0087 (11)	−0.0051 (9)	0.0123 (10)
O5	0.0533 (11)	0.0962 (14)	0.0540 (11)	0.0019 (10)	0.0080 (9)	−0.0174 (9)

### Geometric parameters (Å, °)

**Table:**

C1—O5	1.257 (3)	C13—C14	1.386 (4)
C1—O2	1.258 (3)	C13—C16	1.503 (4)
C1—C2	1.496 (3)	C14—C15	1.371 (3)
C2—C3	1.375 (3)	C14—H14	0.9300
C2—C7	1.383 (3)	C15—H15	0.9300
C3—C4	1.381 (3)	C16—H16A	0.9600
C3—H3	0.9300	C16—H16B	0.9600
C4—C5	1.388 (4)	C16—H16C	0.9600
C4—H4	0.9300	C17—N1	1.339 (3)
C5—C6	1.371 (4)	C17—C18	1.372 (3)
C5—C8	1.512 (4)	C17—H17	0.9300
C6—C7	1.380 (3)	C18—C19	1.364 (4)
C6—H6	0.9300	C18—H18	0.9300
C7—H7	0.9300	C19—C20	1.370 (3)
C8—H8A	0.9600	C19—H19	0.9300
C8—H8B	0.9600	C20—C21	1.374 (3)
C8—H8C	0.9600	C20—H20	0.9300
C9—O3	1.242 (3)	C21—N1	1.346 (3)
C9—O4	1.300 (3)	C21—C21^i^	1.491 (5)
C9—C10	1.472 (3)	Mn1—O5^i^	2.1139 (16)
C10—C11	1.382 (3)	Mn1—O5	2.1139 (16)
C10—C15	1.383 (3)	Mn1—O3^i^	2.1900 (18)
C11—C12	1.374 (4)	Mn1—O3	2.1900 (18)
C11—H11	0.9300	Mn1—N1	2.2607 (19)
C12—C13	1.388 (4)	Mn1—N1^i^	2.2607 (19)
C12—H12	0.9300	O4—H4A	0.8200
			
O5—C1—O2	125.0 (2)	C14—C15—C10	121.1 (2)
O5—C1—C2	117.4 (2)	C14—C15—H15	119.4
O2—C1—C2	117.6 (2)	C10—C15—H15	119.4
C3—C2—C7	117.9 (2)	C13—C16—H16A	109.5
C3—C2—C1	121.0 (2)	C13—C16—H16B	109.5
C7—C2—C1	121.1 (2)	H16A—C16—H16B	109.5
C2—C3—C4	121.3 (2)	C13—C16—H16C	109.5
C2—C3—H3	119.4	H16A—C16—H16C	109.5
C4—C3—H3	119.4	H16B—C16—H16C	109.5
C3—C4—C5	120.7 (3)	N1—C17—C18	123.3 (2)
C3—C4—H4	119.6	N1—C17—H17	118.4
C5—C4—H4	119.6	C18—C17—H17	118.4
C6—C5—C4	117.9 (3)	C19—C18—C17	118.8 (3)
C6—C5—C8	121.5 (3)	C19—C18—H18	120.6
C4—C5—C8	120.6 (3)	C17—C18—H18	120.6
C5—C6—C7	121.4 (3)	C18—C19—C20	118.6 (2)
C5—C6—H6	119.3	C18—C19—H19	120.7
C7—C6—H6	119.3	C20—C19—H19	120.7
C6—C7—C2	120.9 (3)	C19—C20—C21	120.3 (2)
C6—C7—H7	119.6	C19—C20—H20	119.9
C2—C7—H7	119.6	C21—C20—H20	119.9
C5—C8—H8A	109.5	N1—C21—C20	121.3 (2)
C5—C8—H8B	109.5	N1—C21—C21^i^	115.82 (13)
H8A—C8—H8B	109.5	C20—C21—C21^i^	122.83 (16)
C5—C8—H8C	109.5	O5^i^—Mn1—O5	107.91 (10)
H8A—C8—H8C	109.5	O5^i^—Mn1—O3^i^	85.14 (7)
H8B—C8—H8C	109.5	O5—Mn1—O3^i^	91.85 (7)
O3—C9—O4	123.4 (2)	O5^i^—Mn1—O3	91.85 (7)
O3—C9—C10	121.0 (2)	O5—Mn1—O3	85.14 (7)
O4—C9—C10	115.6 (2)	O3^i^—Mn1—O3	174.89 (9)
C11—C10—C15	118.4 (2)	O5^i^—Mn1—N1	162.18 (7)
C11—C10—C9	118.8 (2)	O5—Mn1—N1	89.84 (7)
C15—C10—C9	122.7 (2)	O3^i^—Mn1—N1	96.19 (7)
C12—C11—C10	120.1 (3)	O3—Mn1—N1	87.94 (7)
C12—C11—H11	120.0	O5^i^—Mn1—N1^i^	89.84 (7)
C10—C11—H11	120.0	O5—Mn1—N1^i^	162.18 (7)
C11—C12—C13	122.0 (3)	O3^i^—Mn1—N1^i^	87.94 (7)
C11—C12—H12	119.0	O3—Mn1—N1^i^	96.19 (7)
C13—C12—H12	119.0	N1—Mn1—N1^i^	72.48 (10)
C14—C13—C12	117.1 (3)	C17—N1—C21	117.6 (2)
C14—C13—C16	121.5 (3)	C17—N1—Mn1	124.29 (15)
C12—C13—C16	121.3 (3)	C21—N1—Mn1	117.55 (15)
C15—C14—C13	121.2 (3)	C9—O3—Mn1	127.24 (16)
C15—C14—H14	119.4	C9—O4—H4A	109.5
C13—C14—H14	119.4	C1—O5—Mn1	136.66 (17)

Symmetry codes: (i) −*x*+3/2, −*y*+1/2, *z*.

### Hydrogen-bond geometry (Å, °)

**Table:**

*D*—H···*A*	*D*—H	H···*A*	*D*···*A*	*D*—H···*A*
O4—H4A···O2^i^	0.82	1.68	2.477 (2)	164

Symmetry codes: (i) −*x*+3/2, −*y*+1/2, *z*.
